# Determination and risk assessment of pharmaceutical residues in the urban water cycle in Selangor Darul Ehsan, Malaysia

**DOI:** 10.7717/peerj.14719

**Published:** 2023-02-01

**Authors:** Zarimah Mohd Hanafiah, Wan Hanna Melini Wan Mohtar, Teh Sabariah Abd Manan, Nur Aina Bachi, Nurfaizah Abu Tahrim, Haris Hafizal Abd Hamid, Abdulnoor Ghanim, Amirrudin Ahmad, Nadiah Wan Rasdi, Hamidi Abdul Aziz

**Affiliations:** 1 Department of Civil Engineering, Faculty of Engineering and Built Environment, Universiti Kebangsaan Malaysia, Bangi, Selangor Darul Ehsan, Malaysia; 2 Environmental Management Centre, Institute of Climate Change, Universiti Kebangsaan Malaysia, Selangor Darul Ehsan, Malaysia; 3 Institute of Tropical Biodiversity and Sustainable Development, Universiti Malaysia Terengganu, Kuala Nerus, Terengganu Darul Iman, Malaysia; 4 School of Civil Engineering, Universiti Sains Malaysia, Nibong Tebal, Pulau Pinang, Malaysia; 5 Department of Chemical Sciences, Faculty of Science and Technology, Universiti Kebangsaan Malaysia, Bangi, Selangor Darul Ehsan, Malaysia; 6 Department of Earth Sciences and Environment, Faculty of Science and Technology, Universiti Kebangsaan Malaysia, Bangi, Selangor Darul Ehsan, Malaysia; 7 Department of Civil Engineering, College of Engineering, Najran University, Najran, Saudi Arabia; 8 Faculty of Science and Marine Environment, Universiti Malaysia Terengganu, Kuala Nerus, Terengganu Darul Iman, Malaysia; 9 Faculty of Fisheries and Food Science, Universiti Malaysia Terengganu, Kuala Nerus, Terengganu Darul Iman, Malaysia

**Keywords:** Environmental fate, NSAIDs, Urban water cycle, Ecological risk assessment, Teratogenic index, Risk quotient

## Abstract

The environmental fate of non-steroidal anti-inflammatory drugs (NSAIDs) in the urban water cycle is still uncertain and their status is mainly assessed based on specific water components and information on human risk assessments. This study (a) explores the environmental fate of NSAIDs (ibuprofen, IBU; naproxen, NAP; ketoprofen, KET; diazepam, DIA; and diclofenac, DIC) in the urban water cycle, including wastewater, river, and treated water *via* gas chromatography-mass spectrophotometry (GCMS), (b) assesses the efficiency of reducing the targeted NSAIDs in sewage treatment plant (STP) using analysis of variance (ANOVA), and (c) evaluates the ecological risk assessment of these drugs in the urban water cycle *via* teratogenic index (TI) and risk quotient (RQ). The primary receptor of contaminants comes from urban areas, as a high concentration of NSAIDs is detected (ranging from 5.87 × 10^3^ to 7.18 × 10^4^ ng/L). The percentage of NSAIDs removal in STP ranged from 25.6% to 92.3%. The NAP and KET were still detected at trace levels in treated water, indicating the persistent presence in the water cycle. The TI values for NAP and DIA (influent and effluent) were more than 1, showing a risk of a teratogenic effect. The IBU, KET, and DIC had values of less than 1, indicating the risk of lethal embryo effects. The NAP and DIA can be classified as Human Pregnancy Category C (2.1 > TI ≥ 0.76). This work proved that these drugs exist in the current urban water cycle, which could induce adverse effects on humans and the environment (RQ in high and low-risk categories). Therefore, they should be minimized, if not eliminated, from the primary sources of the pollutant (*i.e*., STPs). These pollutants should be considered a priority to be monitored, given focus to, and listed in the guideline due to their persistent presence in the urban water cycle.

## Introduction

The urban water cycle is heavily infested with emerging contaminants such as pharmaceutical residues, particularly NSAIDs, because of the growing population of sick people who consume the drugs (painkillers, antifungals, antibiotics, antipyretics, analgesics, lipid regulators, antibiotics, antidepressants, chemotherapy agents, antidiabetics, gastric pH regulators and contraceptive drugs) (Hanafiah et al., 2022; Jacob et al., 2021). The Malaysia Statistics on Medicines (MSOM) has reported that the total medicine utilisation in Malaysia increased to 1.2% in 2016. The total medicine consumption in 2015 was 624.90 DDD/1,000 inhabitants/day. It increased to 632.32 DDD/1,000 inhabitants/day in 2016 (MSOM, 2020). The increment in statistics was attributed to the large medicine consumption for chronic diseases such as hypertension, diabetes, cardiovascular disease, and cancer. The consumed drugs go through metabolic detoxification in the human body, releasing 90% of the metabolic waste products (parent and daughter compounds of the pharmaceutical residue) from the total consumption in the form of excreta (sweat, urine, and stool) (Manan, Malakahmad & Sivapalan, 2016; Malakahmad, Abd Manan & Sivapalan, 2015; Subedi et al., 2017). For example, 80% of naproxen does not undergo metabolic activity in the human body and is excreted as a parent compound (Cory et al., 2019). Consequently, these pharmaceutical residues will be released into the wastewater (Mohtar et al., 2017; Zind et al., 2021). The urban water cycle includes the discharge of wastewater from the community (influent), effluent from a sewage treatment plant (STP), receiving surface water (mainly river), and treated water from water treatment plant (WTP) that goes back to the populace *via* the water distribution network (Mohtar et al., 2019). The STP is the central receptacle of pharmaceutical residue and other emerging contaminants (Chopra & Kumar, 2020; Del Pozo & Patel, 2007; Ebele, Abou-Elwafa Abdallah & Harrad, 2017).

Surface water, such as the river widely used as a resource for drinking water, and the presence of micro-pollutants such as pharmaceutical residue affect the sustainable production of clean water for human well-being and subsequently impact the freshwater ecosystem (Khan et al., 2022a). NSAIDs are most frequently detected in the water environment, such as wastewater (Madikizela & Chimuka, 2017), surface water (Tahrim, Abdullah & Farina Abdul Aziz Abstract, 2018), and drinking water (Carmona, Andreu & Picó, 2014) because of the vast consumption to treat fever and relief pain. The STP is not designed to treat and remove pharmaceutical compounds from wastewater (Fröhlich, Foletto & Dotto, 2019). However, these compounds may be chemically- or bio-degraded, absorbed into the sludge, or suspended solids and reduced during treatment (Guerra et al., 2014; Archer et al., 2017). The removal percentages range from 0% to 97% depending on the complexity of the compound (*e.g*., aromatic and low molecular and high molecular weight) (Al Aukidy, Verlicchi & Voulvoulis, 2014; Manan et al., 2019).

Current research focuses on the environmental fate of NSAIDs such as ibuprofen (IBU, 206.28 g/mol), naproxen (NAP, 230.26 g/mol), ketoprofen (KET, 254.28 g/mol), diazepam (DIA, 284.74 g/mol), and diclofenac (DIC, 296.15 g/mol) in the urban water cycle. The generally studied NSAIDs are IBU painkillers for menstrual periods, migraines, toothache, and rheumatoid arthritis; NAP, which is used for reduces inflammation or swelling and treat joint stiffness; KET, which is prescribed for its antipyretic (reduce fever), anti-inflammatory, and analgesic (pain reliever) effects; DIA, which is used as a tranquillizer to reduce anxiety, fear, and tension, as an anticonvulsant muscle relaxant, and a sedative to reduce the states of mental disturbance; and DIC, which is used for an anti-inflammatory to treat menstrual pain, osteoarthritis and rheumatoid arthritis. Although NSAIDs are medicinal drugs approved by the United States Food and Drug Administration (US-FDA), prolonged consumption of NSAIDs is hazardous because they do not cure or alter the root course of the disease and can cause ulcers (Brutzkus, Shahrokhi & Varacallo, 2022). Like other medicine, they reduce the symptoms but are not meant to cure the diseases.

The repetition of exposure to pharmaceutical residue in the cycle seems to continue with no end. It not only is a health hazard (affecting kidneys and liver, antibiotic resistance diseases, and ulcers) but also harms the aquatic environment (Khan et al., 2022b). Although the natural aquatic environment has the natural reclamation mechanism (biological, physical, and chemical processes) for pollutant removal, the complexity of these pharmaceutical residues is chemically designed for treatment purposes and may be fatal to susceptible biological organisms (microbes, plants, fishes, *etc*.). For example, IBU has been reported to affect the survival of Japanese medaka (*Oryzias latipes*) at long-term exposure of 120 days at 100 ng/L (Han et al., 2010), and a low concentration of 250 ng/L can cause endocrine disruption in mussels (*Mytilus galloprovincialis*) (Chopra & Kumar, 2020); DIA inhibits the polyp regeneration of *Hydra vulgaris* (Pascoe, Karntanut & Müller, 2003). It is also a hormone and endocrine disrupting chemical (EDC) because of its effect on the growth and reproduction of fish (Celino-Brady, Lerner & Seale, 2021). The DIA also can induce antibiotic-resistant bacteria in the water environment (Wang et al., 2021). DIA has been reported to have a significant behavioural effect on fish at a medium concentration of 1.0 ± 0.15 µg/L (Lorenzi, Choe & Schlenk, 2016). DIC can cause morphological changes in fish kidneys and have an anti-ovulatory effect on aquatic organisms (Guruge et al., 2019).

Emerging contaminants (ECs) or contaminants of emerging concern (CECs), such as NSAIDs, are newly emerged contaminants that have gained public attention because of their persistence in the environment, risking the security of public health. However, the toxicity profiles of NSAIDs are scarce, and ecological risk assessments for the urban water cycle are yet to be determined. Chemicals with n-octanol/water partition coefficient (log K_ow_, unitless) values of more than 4.5 tend to bio-accumulate (Styszko et al., 2021). The introduction of effluent STP containing pharmaceuticals into the environment presents a toxicology effect on the aquatic living organisms and microorganisms, depending on the type of receiving water, which has a high dilution factor in the open aquatic systems (*i.e*., rivers and seas) and a low dilution factor in a closed aquatic system (*i.e*., lakes). The contaminants can bio-accumulate even at low concentrations and are harmful to the non-targeted organism (Praveenkumarreddy et al., 2021).

Table 1 summarises the pharmaceutical residue (IBU, NAP, KET, DIA, and DIC) in the urban water cycle (influent, effluent, surface water, and treated water). The comparison was among Sweden (Larsson, Al-Hamimi & Jönsson, 2014), Canada (Guerra et al., 2014), South Africa (Madikizela & Chimuka, 2017), Spain (Carmona, Andreu & Picó, 2014; Gracia-Lor et al., 2017; Jurado, Vázquez-Suñé & Pujades, 2021), India (Subedi et al., 2017), Beijing in China (Wang et al., 2017), the United Kingdom (Baker & Kasprzyk-Hordern, 2013), Thailand (Tewari et al., 2013), Italy (Marchese et al., 2003; Zuccato et al., 2000), Serbia (Petrović et al., 2014), France (Togola & Budzinski, 2008), Algeria (Kermia, Fouial-Djebbar & Trari, 2016), and Malaysia (Johor (Yacob et al., 2017), Selangor (Tahrim, Abdullah & Farina Abdul Aziz Abstract, 2018), Putrajaya (Wee et al., 2020)). None of them has published the environmental fate of NSAIDs in the complete urban water cycle thus far.

**Table 1:** The pharmaceutical residue (IBU, NAP, KET, DIA, DIC) in the urban water cycle (influent, effluent, surface water, and treated water).

Country	Influent (ng/L)					Effluent (ng/L)					Ref.
	IBU	NAP	KET	DIA	DIC	IBU	NAP	KET	DIA	DIC	
Sweden	1.67 × 10^4^ to 2.28 × 10^4^	8.80 × 10^3^ to 9.49 × 10^3^	4.40 × 10^4^ to 1.35 × 10^3^		1.55 × 10^3^ to 2.25 × 10^3^	0.00 × 10^0^ to 5.20 × 10^2^	2.20 × 10^2^ to 7.80 × 10^2^	0.00 × 10^0^ to 4.80 × 10^2^		4.00 × 10^2^ to 3.60 × 10^2^	Larsson, Al-Hamimi & Jönsson (2014)
Canada	2.5 × 10^3^ to 4.50 × 10^4^	1.7× 10^3^ to 2.50 × 10^4^				1.6 × 10^1^ to 4.7 × 10^3^	2.00 × 10^1^ to 3.5 × 10^3^				Guerra et al. (2014)
South Africa	6.90 × 10^4^	2.00 × 10^4^			1.60 × 10^4^	2.10 × 10^3^	6.00 × 10^2^			1.4 × 10^3^	Madikizela & Chimuka (2017)
Spain	6.1 × 10^3^ to 1.91 × 10^4^	8.70 × 10^2^ to 2.24 × 10^3^	2.50 × 10^2^ to 4.10 × 10^2^		<LOQ to 7.40 × 10^2^		9.00 × 10^1^ to 2.80 × 10^1^	1.20 × 10^2^ to 4.20 × 10^2^		2.10 × 10^2^ to 6.20 × 10^2^	Gracia-Lor et al. (2017)
Spain											
India				2.30 × 10^1^ to 2.50 × 10^1^					9.50 × 10^0^ to 3.60 × 10^1^		Subedi et al. (2017)
Beijing, China				1.90 × 10^0^ to 6.00 × 10^0^					8.00 × 10^−1^ to 4.70 × 10^0^		Wang et al. (2017)
United Kingdom				6.6 × 10^0^ to 8.00 × 10^0^					5.00 × 10^−1^ to 7.10 × 10^0^		Baker & Kasprzyk-Hordern (2013)
Thailand											
Italy											
Italy											
Serbia											
France											
Algeria											
Johor, Malaysia	1.03 × 10^2^ to 7.69 × 10^2^	1.24 × 10^2^ to 3.75 × 10^2^				n.d. to 2.03 × 10^2^	2.92 × 10^1^ to 8.31 × 10^2^				Yacob et al. (2017)
Selangor, Malaysia											
Putrajaya, Malaysia											
Selangor, Malaysia	** $\sqrt{}$**	$\sqrt{}$	$\sqrt{}$	$\sqrt{}$	$\sqrt{}$	$\sqrt{}$	$\sqrt{}$	$\sqrt{}$	$\sqrt{}$	$\sqrt{}$	Current study

Country	Surface water (ng/L)					Treated water (ng/L)					Ref.
	IBU	NAP	KET	DIA	DIC	IBU	NAP	KET	DIA	DIC	
Sweden											
Canada											
South Africa											
Spain	1.43 × 10^2^		6.13 × 10^1^		2.59 × 10^2^						Jurado, Vázquez-Suñé & Pujades (2021) *Groundwater
Spain						3.90 × 10^1^	1.10 × 10^1^			1.80 × 10^1^	Carmona, Andreu & Picó (2014)
India											
Beijing, China									<LOQ to 1.22 × 10^0^		Zhang et al. (2019)
United Kingdom				8.00 × 10^−1^							Baker & Kasprzyk-Hordern (2013)
Thailand	4.90 × 10^1^	4.10 × 10^1^			1.90 × 10^1^						Tewari et al. (2013)
Italy	1.10 × 10^1^ to 2.00 × 10^1^	1.20 × 10^1^ to 2.20 × 10^1^	2.00 × 10^0^ to 7.00 × 10^0^		5 × 10^0^ to 5.50 × 10^1^						Marchese et al. (2003)
Italy				5.00 × 10^−1^ to 1.20 × 10^0^							Zuccato et al. (2000)
Serbia								1.60 × 10^1^			Petrović et al. (2014)
France						n.d. to 6.00 × 10^−1^	n.d. to 2.00 × 10^−1^			n.d. to 2.5 × 10^0^	Togola & Budzinski (2008)
Algeria								2.73 × 10^2^			Kermia, Fouial-Djebbar & Trari (2016)
Johor, Malaysia											
Selangor, Malaysia			1.10 × 10^2^ to 1.90 × 10^2^								Tahrim, Abdullah & Farina Abdul Aziz Abstract (2018)
Putrajaya, Malaysia										6.46 × 10^0^	Wee et al. (2020)
Selangor, Malaysia	$\sqrt{}$	$\sqrt{}$	$\sqrt{}$	$\sqrt{}$	$\sqrt{}$	$\sqrt{}$	$\sqrt{}$	$\sqrt{}$	$\sqrt{}$	$\sqrt{}$	Current study

Langat River (Sungai Langat) is a 78-km-long river (catchment area: 2,350 km^2^) that flows from the Banjaran Titiwangsa (Titiwangsa ranges) at Gunung Nuang up to the Straits of Malacca. The selected location of Sungai Langat, with a population of most 1 million people, consists of industrial discharge, domestic sewage discharge, construction projects, and farming. Langat River has been classified as an average polluted river with a class III water quality index (WQI) (Heng et al., 2006). Considering the pollution level of Langat River, for the current work, we were choosing one point from Langat River to identify the pharmaceutical residue in the complete urban water cycle, such as influent STP, effluent STP, surface water of Langat River, and treated water from WTP. The environmental fate of NSAIDs in an urban catchment was of paramount importance to be understood, providing an evidence-based way forward in water resources management. The objectives of this research were as follows: (a) to determine the environmental fate of pharmaceutical residue namely IBU, NAP, KET, DIA, and DIC, in the urban water cycle (influent STP, effluent STP, surface water, and treated water), (b) to assess the efficiency of the treatment process by conventional STP, and (c) to evaluate the ecological risk assessment of these drugs in the urban water cycle. This current research presents the concentration of pharmaceutical residue in the urban water cycle from the same catchment and river watershed with justification for why the ecological risk assessment on each water cycle part was not carried out by previous studies.

## Materials and Methods

### Sampling stations and procedure

**Figure 1:**
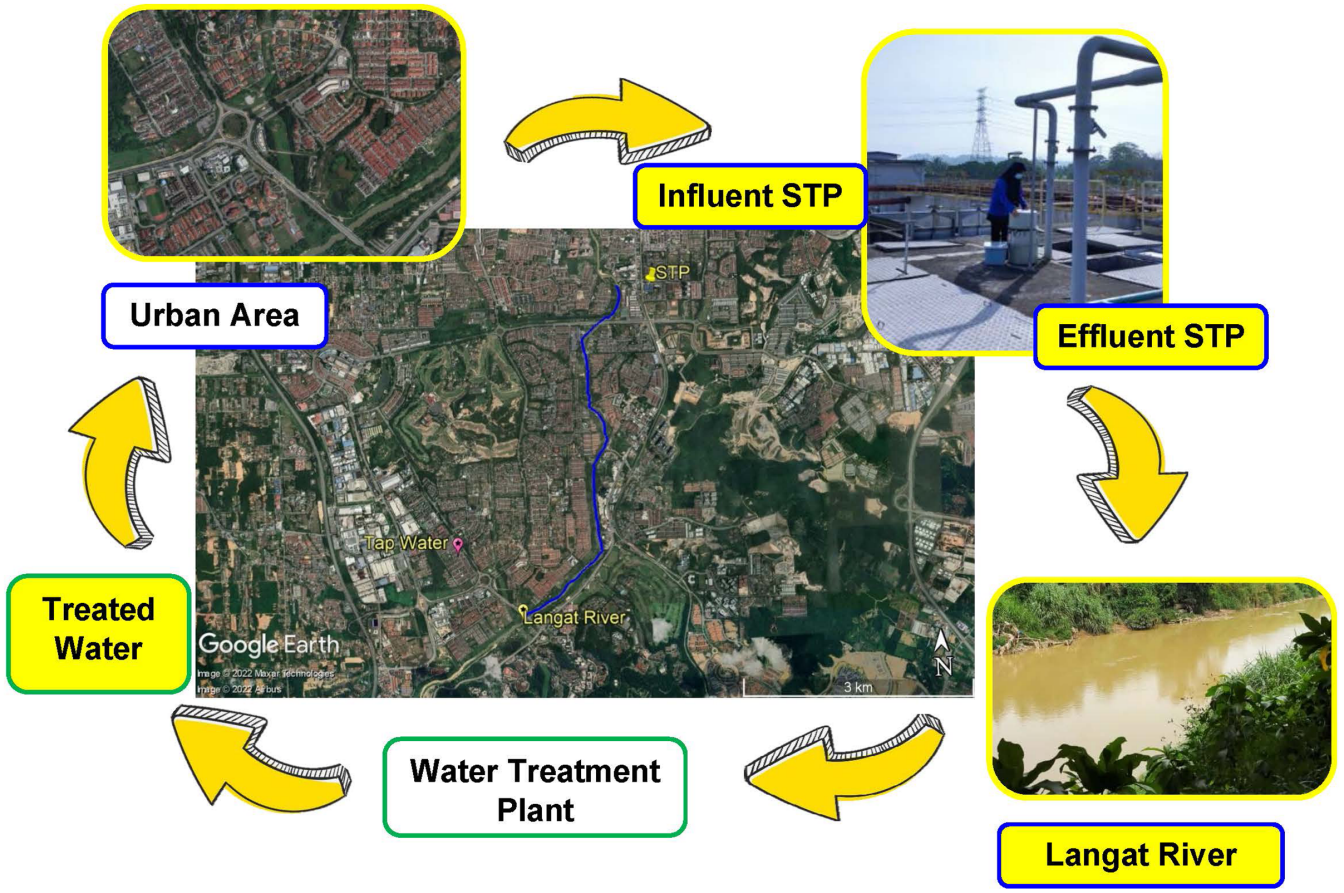
Map of sampling point and urban water cycle (influent STP, effluent STP, Langat River (surface water), and treated water from WTP). Map credit: image © 2022 Maxar Technologies, © Airbus.

The water samples were collected from April to May 2021 from an urban water cycle in Selangor Darul Ehsan, Malaysia. The samples (total 12 and duplicates of each) were taken during three consecutive weekdays for each sampling point (influent and effluent STP week 1 (day 1, 2, and 3); river water week 2 (day 1, 2, and 3); and tap water week 3 (day 1, 2, and 3)). The water receptors in the urban water cycle consisted of STP (influent and effluent), Langat River and housing water supply (treated water from a WTP within the same catchment), as shown in Fig. 1. The STP applied the treatment type of a sequencing batch reactor (SBR) that serves 150,000 population equivalents (PE). The water sampling procedure was carried out using amber glass bottles following the standard method (APHA, 1998). The amber glass bottles (500 mL) were pre-cleaned with dish wash, rinsed with distilled water, and finally rinsed with methanol (MeOH), and dried in the oven for at least 2 h before the sampling activity. All the water samples were kept in a cool box, filtered (0.7-µm glass fibre filter (GF/F); Whatman, Maidstone, UK), adjusted to pH 3 using HCl (1 M), and stored in the cold room (4 °C). The samples were extracted within 48 h after sampling.

### Chemicals and reagents

The chemicals used were HPLC-grade MeOH (Thermo Fisher Scientific, Seoul, Korea), AR-grade ethyl acetate (EA) (Systerm, Shah Alam, Malaysia), and hydrochloric acid (HCl) 37% (Merck, Darmstadt, Germany). The ultra-pure water (UPW) with a resistivity of 18.2 MΩ cm was obtained from the Smart N system (Heal Force, Shanghai, China). High-purity (>98%) individual standards for the targeted compounds IBU, NAP, KET, and DIC were purchased from Alfa Aesar (UK) and DIA from Sigma Aldrich (St. Louis, MO, USA) (Table S1). The MSTFA reagent (Sigma Aldrich, St. Louis, MO, USA) was used for derivatization. The stock solutions were prepared by dissolving each standard compound in 100 mL of MeOH (1 mg/mL). Mix working solutions were prepared by spiking each standard compound in MeOH at the required final concentration to prepare the external standard. All of the standard and stock solutions were stored at 4 °C.

### Solid phase extraction (SPE) method

The SPE protocol was based on a previous method described by Hanafiah et al. (2022). The SPE cartridges were Oasis HLB (6 cc, 200 mg; Waters, Milford, MA, USA). The hydrophilic-lipophilic balance (HLB) cartridge was first conditioned using 3 mL EA, 3 mL MeOH and 3 mL UPW (adjusted to pH 3 using 1 M HCl). Then, 100 mL of the filtered samples were passed through the cartridge and rinsed with 10 mL of mixed MeOH and UPW with a ratio 40:60 (v/v). The cartridge was vacuum dried for 20 to 30 min and the targeted analyte was eluted with 10 mL of mixed EA and acetone in the ratio of 50:50 (v/v) in 10 mL vials. The sample was then gently dried under a stream of purified nitrogen gas to approximately 2 mL and re-dissolved by adding 100 µL of EA. Finally, the samples were derivatized with 30 µL of MSTFA and incubated at 80 °C for 30 min.

### Standards, quality assurance, and calibration curves

The calibration curve for each of the targeted analytes was established on the basis of the external standard of concentration between 0.5 to 5 mg/L. The calibration curve shows satisfactory linearity with the correlation coefficient for IBU (*R*^*2*^= 0.9844), NAP (*R*^*2*^= 0.9607), KET (*R*^*2*^= 0.9889), DIC (*R*^*2*^= 0.9992) and DIA (*R*^*2*^= 0.9755) (Table S2). The recovery experiments were based on 100 mL of the effluent samples pre-spike and post-spike (treated the same as the sample in the SPE method) with known concentrations for all of the target analytes. The percentage recovery was then calculated using Eq. (1).

(1) $$RE\; \left( \% \right) = \displaystyle{{Area\; of\; Pre - spike\; Sample} \over {Area\; of\; Post - spike\; Sample}}\; \times 100$$

The recovery of the targeted analyte in the extraction experiment was IBU (99.1%), NAP (97.7%), KET (138.2%), DIC (69.1%), and DIA (74.9%). The acceptable range for recovery was from 50% to 120%, and the coefficient of variation value was less than 20% (Abd Manan et al., 2021). The limit of detection (LOD) and the limit of quantification (LOQ) were calculated on the basis of 3.3 times and ten (10) times of the standard ratio deviation to the slope of the calibration curve (σ/s) accordingly (Gupta, 2014). The LOD values were 4.337 × 10^5^ ng/L for IBU, 3.854 × 10^5^ ng/L for NAP, 6.754 × 10^5^ ng/L for KET, 3.54 × 10^5^ ng/L for DIC, and 6.865 × 10^5^ ng/L for DIA. The LOQ values were 1.314 × 10^6^ ng/L for IBU, 1.168 × 10^6^ ng/L for NAP, 2.046 × 10^6^ ng/L for KET, 1.072 × 10^6^ ng/L for DIC, and 2.080 × 10^6^ ng/L for DIA. Initially, the calibration curves were presented in milligrams per litre (mg/L), and the back calculation result for the quantification of the analyte in the sample produced a reading in mg/L units. To standardise the discussion, all of the values were presented in units of nanograms per litre (ng/L).

### Gas chromatography-mass spectrometry condition

The targeted analytes were analysed using GCMS Agilent 6890 N with the GC system connected to the MSD 5975 detector (Agilent Technologies, Santa Clara, CA, USA). The setting of the purified helium gas (>99.99%) was set at a flow of 1.3 mL/min and the separation of the chromatogram was achieved using capillary column type HP-5 MS (30 m × 0.32 mm × 0.25 µm) (J.W. Scientific, Folsom, CA, USA). Then, 1 µL of the sample was injected in the splitless mode at 250 °C by using the Agilent 7683B automatic liquid sampler injector. The GC oven temperature was set at an initial temperature of 70 °C for 2 min; the temperature was then increased to 280 °C at the rate of 15 °C/min and held for 2 min. The targeted analyte was identified using MS software NIST version 5.5 under the selected ion monitoring (SIM) mode.

### Statistical analysis

A comparison between each group of NSAIDs found in the water cycle outcomes was performed by analysis of variance (ANOVA) using the SPSS statistical program. Pearson’s correlation analysis was conducted to evaluate the relationship between the water cycle points. The values of the correlation coefficient obtained ranged from −1 (indication of a negative linear correlation) to 0 (indication of no linear relationship) to +1 (indication of a positive linear correlation) (Kim et al., 2021).

### Ecological risk assessment

Any drugs having n-octanol-water partition coefficient (log K_ow_) values of more than 4.5 should be monitored for their persistency (exist in prolonged period) and toxicity (OSPAR, 1992). These high log K_ow_ value drugs tend to bioaccumulate in organic matter such as soils or sediments (ChemSafetyPro, 2021). Although the log K_ow_ values for the drugs in this study (IBU 3.97, NAP 3.18, KET 2.66, DIA 2.85, and DIC 1.90) were less than 4.5, ecological risk assessment was used to estimate their risk to the aquatic ecosystem. The RQ calculation is shown in Eq. (2) (European Medicines Agency, 2006; Bouissou-Schurtz et al., 2014; Li et al., 2021).

(2) $$R{Q_{MEC}} = \; \displaystyle{{MEC} \over {PNEC}}$$

where MEC denoted the measured environmental concentration and PNEC represents the predicted no effect concentration.

The predicted no effect concentration (PNEC) values were estimated using the lowest acute toxicity data from the literature (50% effective concentration, EC_50_; or 50% lethal concentration, LC_50_) for the first trophic level (algae, invertebrates, plants, and fish) and applying an assessment factor of 1,000 (European Commission, 2003; Amiard & Amiard-Triquet, 2015; Hanafiah et al., 2022).

The RQMEC represents a real risk (European Medicines Agency, 2006). The RQ values of more than 1 indicate a high ecological risk, values of more than 0.1 and less than 1 (0.1 < RQ < 1) show a medium ecological risk, and values less than 0.1 (RQ < 0.1) show a low ecological risk to aquatic organisms (European Commission, 2003; Huang et al., 2019).

The EC and LC values are listed in the literatures (Table 2). The teratogenicity index (TI) was calculated using the quotient of LC_50_ and EC_50_ (Eq. (3)). Any TI value greater than 1 was an indication of the teratogenic effect, while any TI value below 1 represented that the substance produced embryo lethal effects (Nordberg et al., 2009; Weigt et al., 2011; Lee et al., 2013).

(3) $$TI = \displaystyle{{L{C_{50}}} \over {E{C_{50}}}}$$

**Table 2:** Toxicological dose descriptors for IBU, NAP, KET, DIA, and DIC based on different aquatic organisms (fish, algae, crustaceans, and bacteria).

No.	Aquatic organisms	NSAIDs	Unit	Toxicological dose descriptors						PNEC	Species of aquatic organisms	Ref.
				LC50	Species of aquatic organisms	Ref.	EC50	Species of aquatic organisms	Ref.			
1.	Fish	**IBU**	**µg/L**	**8.06**	**Embryotoxicity on *Danio rerio***	Han et al. (2010)	**2.85**	**Embryotoxicity on *Danio rerio***	Han et al. (2010)	**1.4** **(7 d)**	***Danio rerio* for mortality**	Huang et al. (2018)
		NAP	mg/L	52 (96 h)	*Oncorhynchus mykiss*	OECD (2019)	*	*		1.0	Fish early-life stage toxicity with *Pimephales promelas*	OECD (2013)
		**KET**	**mg/L**	**6.11** **(96 h)**	***Danio rerio***	Praskova et al. (2011)	**1.91**	**Embryotoxicity on *Danio rerio***	Ben Chabchoubi et al. (2022)	**6.25** **(9 d)**	***Danio rerio* (hatch, mortality, growth)**	Watanabe et al. (2016)
		DIA	mg/L	12.7 (96 h)	*Gambusia holbrooki*	Sanderson et al. (2003)	*	*		*	*	
		DIC	mg/L	6.11 (96 h)	Embryotoxicity on *Danio rerio*	Praskova et al. (2011)				0.32 (34 d)	*Danio rerio* for survival	dos Santos et al. (2021) and Fass.se (2022)
2.	Algae	IBU	mg/L	*	*		342.2 (3 d)	*Desmodesmus subspicatus*	Cleuvers (2004)	10 (3 d)	*Pseudokirchneriella subcapitata*	Huang et al. (2018) (µg/L)
		NAP	mg/L	*	*		39 (72 h)	*Pseudokirchinella subcapitata*,	OECD (2011)	6.2 (72 h)	*Desmodesmus subspicatus*,	OECD (2011)
		KET	mg/L	*	*		0.24 (96 h)	*Pseudokirchneriella subcapitata*	Mennillo et al. (2018)	9.94 (72 h)	*Pseudokirchneriella subcapitata*	Watanabe et al. (2016)
		DIA	mg/L	16.5 (96 h)	*Tetraselmis chuii*	Nunes, Carvalho & Guilhermino (2005)	*	*		1.387 (72 h)	*Pseudokirchneriella subcapitata*	Jacob et al. (2020)
		DIC	mg/L	*	*		72 (3 d)	*Desmodesmus subspicatus*	Cleuvers (2003)	10 (96 h)	*Pseudokirchneriella subcapitata*	Ferrari et al. (2003)
3.	Crustaceans	**IBU**	**µg/L**	**128,500 (2 d)**	***Daphnia magna***	Huang et al. (2018)	**11,500 (2 d)**	***Daphnia magna***	Huang et al. (2018)	**33,300** **(21 d)**	***Daphnia magna*, for mortality**	Huang et al. (2018)
		**NAP**	**mg/L**	**110** **(96 h)**	***Gammarus pulex***	AstraZeneca (2005)	**174** **(48 h)**	***Daphnia magna***	Cleuvers (2003)	**12** **(96 h)**	***Gammarus pulex***	AstraZeneca (2005)
		KET	mg/L	*	*		24.84	*Ceriodaphnia silvestrii*	Lopes Caldas, Moreira & Novelli (2021)	22.5 (6–8 d)	*Ceriodaphnia dubia*	Watanabe et al. (2016)
		**DIA**	**mg/L**	**12.2** **(48 h)**	***Artemia parthenogenetica***	Nunes, Carvalho & Guilhermino (2005)	**14.1**	***Daphnia magna***	Calleja, Persoone & Geladi (1994)	**5.0**	***Mysidopsis juniae***	Da Silva et al. (2020)
		**DIC**	**mg/L**	**80.1** **(2 d)**	***Daphnia magna***	dos Santos et al. (2021) and Han et al. (2010)	**22.43 (2 d)**	***Daphnia magna***	Ferrari et al. (2003)	**1** **(7 d)**	***Ceriodaphnia dubia*, for reproduction**	Ferrari et al. (2003)
4.	Bacteria	IBU		*	*		*	*		*	*	
		NAP	mg/L	*	*		12.3	*Anabaena; flos-aquae*	OECD (2011)	4.0	*Anabaena flos-aquae*	OECD (2011)
			ng/L	*	*		264	Microcosms (dominance of Alpha- and Gamma-Proteobacteria)	Grenni et al. (2013)	*	*	
		KET		*	*		*	*		*	*	
		DIA		*	*		*	*		*	*	
		DIC		*	*		*	*		*	*	

**Note:**

Bold text: Complete list from literatures (LC50, EC50, and PNEC); *: list not completed.

## Results and discussion

### Environmental fate of pharmaceutical residue in urban water cycle

**Figure 2:**
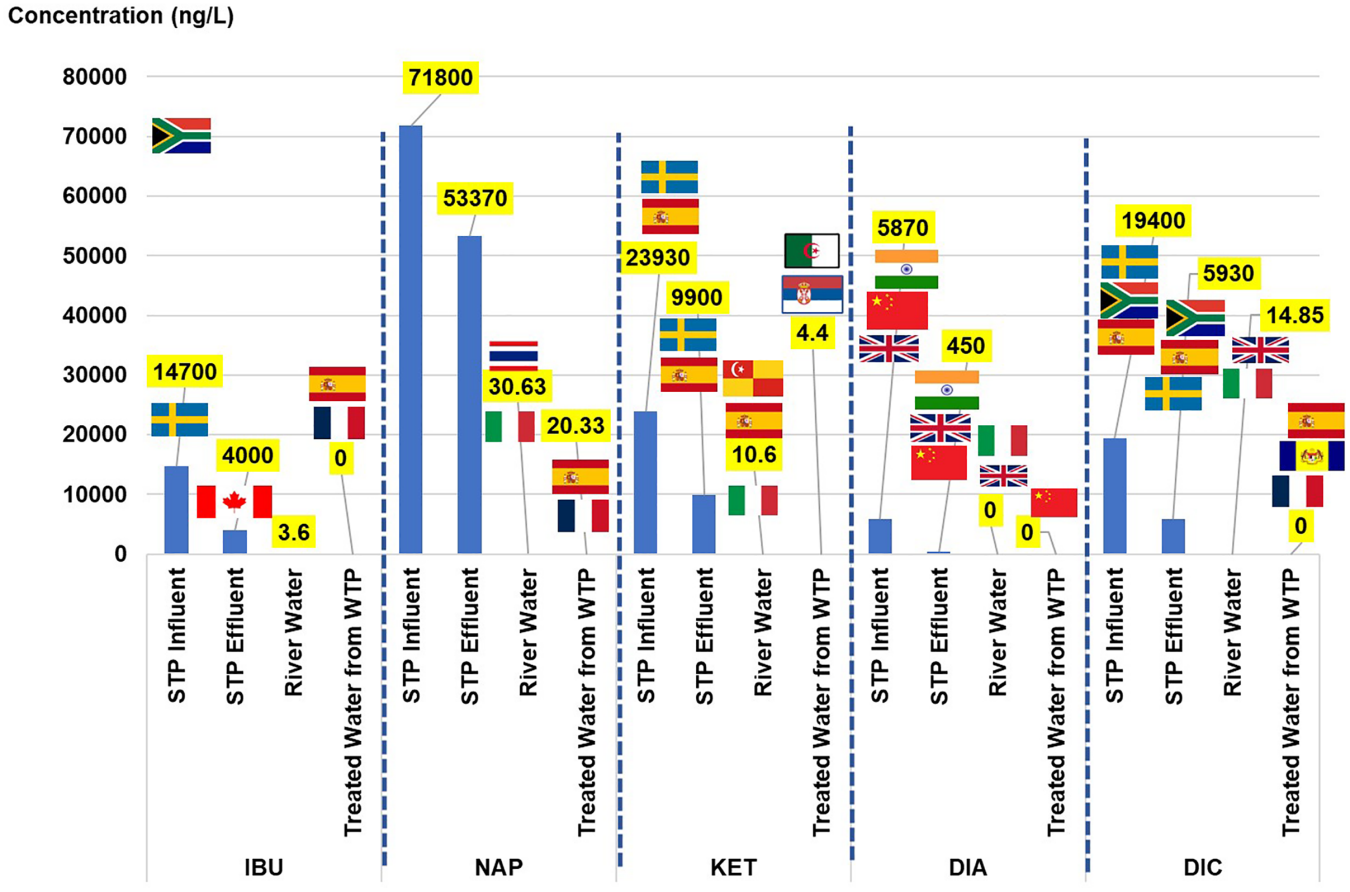
Comparison of NSAIDs in the current study to other countries in the urban water cycle.

NSAIDs can be classified into two therapeutic groups: anti-inflammatory (IBU, NAP, KET, and DIC) and psycholeptics (DIA). Both of the groups have been listed in the top 50 utilised drugs in 2015 and 2016, according to MSOM 2015–2016. To provide an overview of the NSAID scenario, the results were simultaneously presented with the values of the toxicological dose descriptors and the risk quotient. The discussion started with the concentration of each NSAID element found in the urban water cycle and its comparison with the values obtained from other countries based on the extant literature. The concentration of NSAIDs (ng/L) and their toxicological dose descriptors in the urban water cycle are listed in Table 3. The visual comparison of NSAIDs in the current study to other countries (as listed in Table S3) in the urban water cycle is shown in Fig. 2.

**Table 3:** The concentration of NSAIDs (ng/L) and their toxicological dose descriptors in the urban water cycle.

NSAIDs	Urban water cycle	Concentration (ng/L)	Toxicological dose descriptors			Risk quotient	Aquatic organisms
			RQ LC50	RQ EC50	TI		
IBU	Influent STP	1.47 × 10^4^	1.82 × 10^0^	5.16 × 10^0^	3.54 × 10^−1^	1.05 × 10^1^	Fish
	Effluent STP	4.00 × 10^3^	4.96 × 10^−1^	1.40 × 10^0^	3.54 × 10^−1^	2.86 × 10^0^	
	Surface water	3.6 × 10^0^	4.47 × 10^−4^	1.26 × 10^−3^	3.54 × 10^−1^	3.00 × 10^−3^	
	Treated water from WTP	0.00 × 10^0^	0 × 10^0^	0 × 10^0^	0 × 10^0^	0 × 10^0^	
NAP	Influent STP	7.18 × 10^4^	6.53 × 10^−4^	4.13 × 10^−4^	1.58 × 10^0^	5.98 × 10^−3^	Crustacean
	Effluent STP	5.34 × 10^4^	4.85 × 10^−4^	3.07 × 10^−4^	1.58 × 10^0^	4.45 × 10^−3^	
	Surface water	3.06 × 10^1^	2.78 × 10^−7^	1.76 × 10^−7^	1.58 × 10^0^	2.55 × 10^−6^	
	Treated water from WTP	2.03 × 10^1^	1.85 × 10^−7^	1.17 × 10^−7^	1.58 × 10^0^	1.69 × 10^−6^	
KET	Influent STP	2.39 × 10^4^	3.92 × 10^−3^	1.25 × 10^−2^	3.13 × 10^−1^	3.83 × 10^−3^	Fish
	Effluent STP	9.90 × 10^3^	1.62 × 10^−3^	5.18 × 10^−3^	3.13 × 10^1^	1.58 × 10^−3^	
	Surface water	1.06 × 10^1^	1.73 × 10^−6^	5.55 × 10^−6^	3.13 × 10^−1^	1.70 × 10^−6^	
	Treated water from WTP	4.4 × 10^0^	0 × 10^0^	0 × 10^0^	0 × 10^0^	0 × 10^0^	
DIA	Influent STP	5.87 × 10^3^	4.81 × 10^−4^	4.16 × 10^−4^	1.16 × 10^0^	1.17 × 10^−3^	Crustacean
	Effluent STP	4.50 × 10^2^	3.69 × 10^−5^	3.19 × 10^−5^	1.16 × 10^0^	4.50 × 10^−4^	
	Surface water	0.00 × 10^0^	0 × 10^0^	0 × 10^0^	0 × 10^0^	0 × 10^0^	
	Treated water from WTP	0.00 × 10^0^	0 × 10^0^	0 × 10^0^	0 × 10^0^	0 × 10^0^	
DIC	Influent STP	1.94 × 10^4^	2.42 × 10^−4^	8.65 × 10^−4^	2.80 × 10^−1^	1.94 × 10^−2^	Crustacean
	Effluent STP	5.93 × 10^3^	7.40 × 10^−5^	2.64 × 10^−4^	2.80 × 10^−1^	5.93 × 10^−3^	
	Surface water	1.49 × 10^1^	1.85 × 10^−7^	6.62 × 10^−7^	2.80 × 10^−1^	1.49 × 10^−5^	
	Treated water from WTP	0.00 × 10^0^	0 × 10^0^	0 × 10^0^	0 × 10^0^	0 × 10^0^	

The sources of pharmaceuticals in the water cycle can be considered diverse because of their contribution from pharmaceutical industries, hospitals, direct disposal from remaining medicine, aquaculture, and agriculture. Wastewater from the STPs can be considered one of the primary sources as they are the centre of the water collection from the community. The load of NSAIDs in the STPs varies depending on the treatment plant’s efficiency (Singh et al., 2019). The current study detected the IBU in the influent, effluent, and river water but not in the treated water. Although 90% of the IBU would be metabolised in the human body after 24 h upon oral administration, approximately 1% to 12% of the drug will be excreted as the parent compound and major metabolites (carboxy-ibuprofen and 2-hydroxy-ibuprofen) in urine and could accumulate in the plant as high consumption from the community (Mazaleuskaya et al., 2015; Chopra & Kumar, 2020; Escolà Casas et al., 2021).

The IBU concentrations in the influent were consistently detected in Sweden, Canada, and Spain (range 6.1 × 10^3^ to 4.50 × 10^4^ ng/L), with the highest detected in South Africa (6.90 × 10^4^ ng/L), and Johor reported the lowest concentration (1.03 × 10^2^ to 7.69 × 10^2^ ng/L). The IBU concentration in the effluent of the current study was consistent with that of Canada and South Africa (1.60 × 10^1^ to 4.7 × 10^3^ ng/L) and slightly higher than those detected in Sweden and Johor (2.03 × 10^2^ to 5.20 × 10^2^ ng/L). The current study’s concentration in the river water was the lowest compared to those reported in Spain, Thailand, and Italy, ranging between 1.10 × 10^1^ and 1.43 × 10^2^ ng/L. The IBU was not detected in the treated water in the current study, unlike those reported in Spain and France (6.00 × 10^−1^ to 3.90 × 10^1^ ng/L).

Two NSAIDs (NAP and KET) were present in all of the water samples in the water cycle. The NAP concentration in the influent and the effluent was the highest as compared to the others reported in the literature, Johor, Sweden, Canada, South Africa, and Spain, in the range of 1.24 × 10^2^ to 3.75 × 10^4^ ng/L, and 0.292 × 10^1^ to 3.5 × 10^3^ ng/L of the influent and the effluent, respectively. The presence of NAP in the river water was comparable to that in Thailand, Serbia, and Italy, ranging from 2.20 × 10^1^ ng/L to 7.42 × 10^1^ ng/L. In the treated water, the current study reported the highest concentration as compared to Spain and France in the range of 1.10 × 10^1^ to 2.00 × 10^−1^ ng/L. The parent compound excretion of NAP after oral consumption was approximately 0.6% to 15% through bile and urine (Escolà Casas et al., 2021). The presence of NAP in the water sample (river water) was an indication of the commonly used drug in the community and its persistence in the treatment process in water treatment because of the detection in the treated water.

Comparable to NAP, the KET was the highest observed in both the influent and the effluent among the values reported in the literature, Sweden, and Spain, in the range 2.50 × 10^2^ to 1.35 × 10^3^ ng/L and not detected to the 4.80 × 10^2^ ng/L level, respectively. Even so, in the case of the river water, the concentration observed present study was comparable to that in Italy (2 × 10^0^ to 7 × 10^0^ ng/L), and lower than that in Spain and Selangor (6.13 × 10^1^ to 1.90 × 10^2^ ng/L). The presence of KET in the treated water was lower than that in Serbia and Algeria (1.6 × 10^1^ to 2.43 × 10^2^ ng/L). The KET was among the most frequently prescribed analgesic in Malaysian public hospitals from 2010 to 2016 (Zin et al., 2018). The KET also has 80% excretion (Kermia, Fouial-Djebbar & Trari, 2016), making it easy to detect at high concentrations in the water bodies.

The DIA in the current study was detected in both the influent and the effluent STP, and the river water but not in the treated water. The concentration of DIA in the influent and the effluent STP was higher than that in other countries such as India, Beijing, and the United Kingdom, with their concentration ranging from 1.9 × 10^0^ to 2.50 × 10^1^ ng/L, and 0.50 × 10^1^ to 3.60 × 10^1^ ng/L, respectively. The DIA concentration in the river water presented in the current study was low as compared to that in the United Kingdom and Italy (5.00 × 10^−1^ to 1.2 × 10^0^ ng/L). The DIA in treated water was not detected in the current study but was reported in Beijing (1.22 × 10^0^ ng/L) and Italy (2.00 × 10^−1^ ng/L) at low concentrations. In 2016, DIA was reported to be the highest prescribed (in the private and public sectors) for anxiety disorder, and the trend increased from 0.2366 to 0.2903 DDD/1000 inhabitants/day. In all, 6.4% to 9.0% of the DIA would be excreted through metabolic activities in the human body (Wang et al., 2017).

The DIC concentrations were present in the influent, effluent, and river water and not in treated water. The DIC concentration presented in influent and effluent of the current study was comparable to South Africa (influent: 1.60 × 10^4^ ng/L, effluent: 1.4 × 10^3^ ng/L), and lower concentration was detected in Sweden and Spain (influent: 7.40 × 10^2^ to 3.60 × 10^1^ ng/L, effluent: 4.00 × 10^1^ ng/L to 6.20 × 10^2^ ng/L). The DIC concentration in the river water presented in the current study was lower than that in Spain, Thailand, and Italy (5.0 × 10^0^ to 2.59 × 10^2^ ng/L). Although DIC was not detected in the treated water of the current study, the drugs were reported in the treated water from France, Spain, and Putrajaya, Malaysia, in low concentrations between 2.50 × 10^0^ to 1.80 × 10^1^ ng/L. In all, 15% DIC would be excreted through the metabolic activities in the human body (Alder et al., 2006).

The compound’s concentration in the influent was higher than in the effluent, showing that the compound reduced from 99.97% up to 100% during the treatment process from the influent to the treated water stage. The IBU, DIA, and DIC achieved 100% removal, while 99.97% and 99.98% for NAP and KET, respectively. Although the percentage differences of NSAIDs achieved 99.97% to 100% of removal, the remaining concentrations of NSAIDs in the treated water were still significant at the nanograms per litre scale for NAP (20.33 ng/L), and KET (4.4 ng/L). The 100% removal for IBU, DIA, and DIC was considered complete removal on the basis of the “not detected” status. However, the IBU, DIA, and DIC could undergo a multiset of degradation (*e.g*., decarboxylation, hydroxylation, and oxidation) during the treatment in WTP, forming ranges of chlorinated disinfection by-products (Wang et al., 2014; Huang et al., 2021) and these parent compounds were not detected in the original form.

A similar reduction pattern was reported in Thailand (Tewari et al., 2013), South Africa (Madikizela & Chimuka, 2017), India (Subedi & Kannan, 2015), and Spain (Carmona, Andreu & Picó, 2014). In Thailand, 13% to 95% of IBU, NAP, and DIC were removed from five STPs in Bangkok, Thailand (Tewari et al., 2013). South Africa also showed a high percentage removal of IBU, NAP, and DIC (92% to 97%) (Madikizela & Chimuka, 2017). In India, the negative removal of DIA was reported from an STP-type conventional aerobic biological treatment, while 95% of DIA was removed from STP-type up-flow anaerobic sludge blanket reactor (UASBR) (Subedi & Kannan, 2015). Overall, different types of the biological treatment processes have different efficiencies of NSAID removal (Carmona, Andreu & Picó, 2014).

### Statistical analysis

The ANOVA analysis of NSAIDs in the urban water cycle was based on three (3) models: influent *vs* effluent, effluent *vs* river water, and river water *vs* treated water (as shown in Table S4). ANOVA is used to determine the nature of the relationship between one dependent variable (y-axis) and a series of independent variables (x-axis). H_0_ indicates that the population means are equal, showing no difference observed. However, the differences between groups are statistically significant at α less than 0.05. The ANOVA coefficients (significant F change) were 0.001 (influent *vs* effluent), 0.026 (effluent *vs* river water), and 0.044 (river water *vs* treated water).

The steadily increasing trends of prescription NSAIDs to patients in Malaysia (Zin et al., 2018) have led to a high concentration detection in the influent. Every reaction consists of substrates (introduced contaminants in the urban water cycle such as NSAIDs) and products (by-products due to chemical, physical, and biological degradation). The NSAID concentration in the influent *vs* effluent model was significantly different, showing that the STP treatment processes achieved a certain efficiency level for NSAID removal. The typical layout of the STP treatment process consists of a preliminary treatment (screens and grit removal), primary clarifier (solids removal through gravitational settling), biological treatment (aeration tank that will biodegrade high energy molecules with the help of micro-organisms such as bacteria, fungus, rotifers, and protozoa), a secondary clarifier (removal of nitrogen *via* nitrification and denitrification; and phosphorus *via* precipitation), and anaerobic treatment of the sludge (biochemical process mediated by specialised microorganisms *via* hydrolysis, fermentation, and methanogenesis) (Mihelcic, Zimmerman & Zhang, 2002). The primary treatment removes 60% of the suspended solids, 30% of the biochemical oxygen demand, and 20% of the phosphorus. Furthermore, the IBU, NAP, KET, DIA, and DIC showed 72.8%, 25.6%, 58.6%, 92.3% and 69.4% removal accordingly after the treatment processes in the STP.

Next, the NSAID concentration in the effluent *vs* river water model noted a significant difference because of the aqueous dilution from the effluent in the open aquatic ecosystem (Langat River), as well as the reclamation process by the physical factor (irradiation from sunlight) and biodiversity of the aquatic organisms such as plants, fish, fungi, bacteria (Chopra & Kumar, 2020), and microcosm (Grenni et al., 2013) in the surrounding ecosystem (Malakahmad, Abd Manan & Sivapalan, 2015).

The NSAID concentration in the river water *vs* treated water model also showed a statistically significant difference indicating that the treatment processes in the WTP had reduced the initial concentration in the river water (before treatment). The treatment stages of WTP consisted of (1) collection (influent, raw water); (2) screening of particulate material; (3) pH control (addition of 98% of lime soda ash, Na_2_CO_3_) and disinfection (chlorination using free chlorine, combined chlorine or chlorine dioxide); (4) coagulation (addition of coagulants such as aluminium sulphate), flocculation from rapid mix and sedimentation; (5) granular filtration (sand filter bed, to remove pathogens such as *Gardia* and *Cryptosporidium*); (6) disinfection (chlorination); (7) clear-well storage; and (8) distribution network system (effluent and treated water) (Mihelcic, Zimmerman & Zhang, 2002). The problem arose from stage 3 to 6 of the chlorinated by-products of the contaminants.

The NSAIDs consist of benzene rings (IBU: 1 ring, NAP: 2 rings, KET: 2 rings, DIA: 3 rings, and DIC: 2 rings) (National Library of Medicine, 2022), making them hard to degrad *via* a conventional treatment process in an STP (Arany et al., 2013). The IBU is easily degraded because it has only one benzene ring. It is susceptible to oxidation and photo-oxidation processes. It can produce 13 degradation products, including seven newly reported compounds, namely hydrotropic acid, 4-ethylbenzaldehyde, 4-(1-carboxyethyl)benzoic acid, 1-(4-isobutylphenyl)-1-ethanol, 2-(4-(1-hydroxy-2-methylpropyl)phenyl)propanoic acid, 1-isobutyl-4-vinylbenzene, and 4-isobutylphenol (Caviglioli et al., 2002). The NAP can only be metabolized *via* photolysis or an advanced oxidation process producing two primary photolysis by-products, namely 1-(6-methoxy-2-naphthyl) ethanol (NAP-PT1) and 2-acetyl-6-methoxynaphthalene (NAP-PT2) (Cory et al., 2019). The KET can be mineralized *via* electro-oxidation, producing metabolites such as 3-hydroxybenzoic acid, pyrogallol, catechol, benzophenone, benzoic acid, and hydroquinone (Feng et al., 2014). The DIA consists of six transformation pathways for its complete degradation producing 70 by-products (Yang et al., 2020). Yang et al. (2020) reported that UV/chlorine (62.1% removal in 90 min) and sunlight/chlorine (53.1% removal in 90 min) processes could assist in completing the degradation of DIA at a faster rate assisted by the reactive species of hydroxyl radicals. The transformation by-products were less toxic than the parent compound (Yang et al., 2020). The DIC could be rapidly degraded by direct photolysis under normal environmental conditions or other advanced oxidation processes, producing 18 intermediates at different pathways (Pérez-Estrada et al., 2005).

The exposure to direct sunlight in the open surface water body explained the almost complete degradation of NSAIDs in the river water (Langat River) and the treated water of WTP. The river water was still contaminated with NSAIDs at the nanoscale (IBU: 3.6 × 10^0^ ng/L, NAP: 3.06 × 10^1^ ng/L, KET: 1.06 × 10^1^ ng/L, DIA: 0.00 × 10^0^* ng/L (not detected), and DIC: 1.49 × 10^1^ ng/L). The IBU, DIA, and DIC were not detected in the treated water. However, the NAP (2.03 × 10^1^ ng/L) and KET (4.4 × 10^0^ ng/L), still existed at low concentrations in the treated water. In addition, there is still a probability of their metabolite existence in both water receptors (river water and treated water) (Arany et al., 2013; Cory et al., 2019).

Pearson’s correlation on NSAIDs in the urban water cycle is shown in Table S5. In descending sequence, the positive linear correlations were observed on influent *vs* effluent (0.994), effluent *vs* treated water (0.992), influent *vs* treated water (0.982), influent *vs* river water (0.950), effluent *vs* river water (0.921), and river water *vs* treated water (0.888). The results showed that good predictions of NSAIDs in the urban water cycle could be made by pairing variables accordingly. Further spatial-temporal analysis is highly recommended.

### Ecological risk assessment

**Figure 3:**
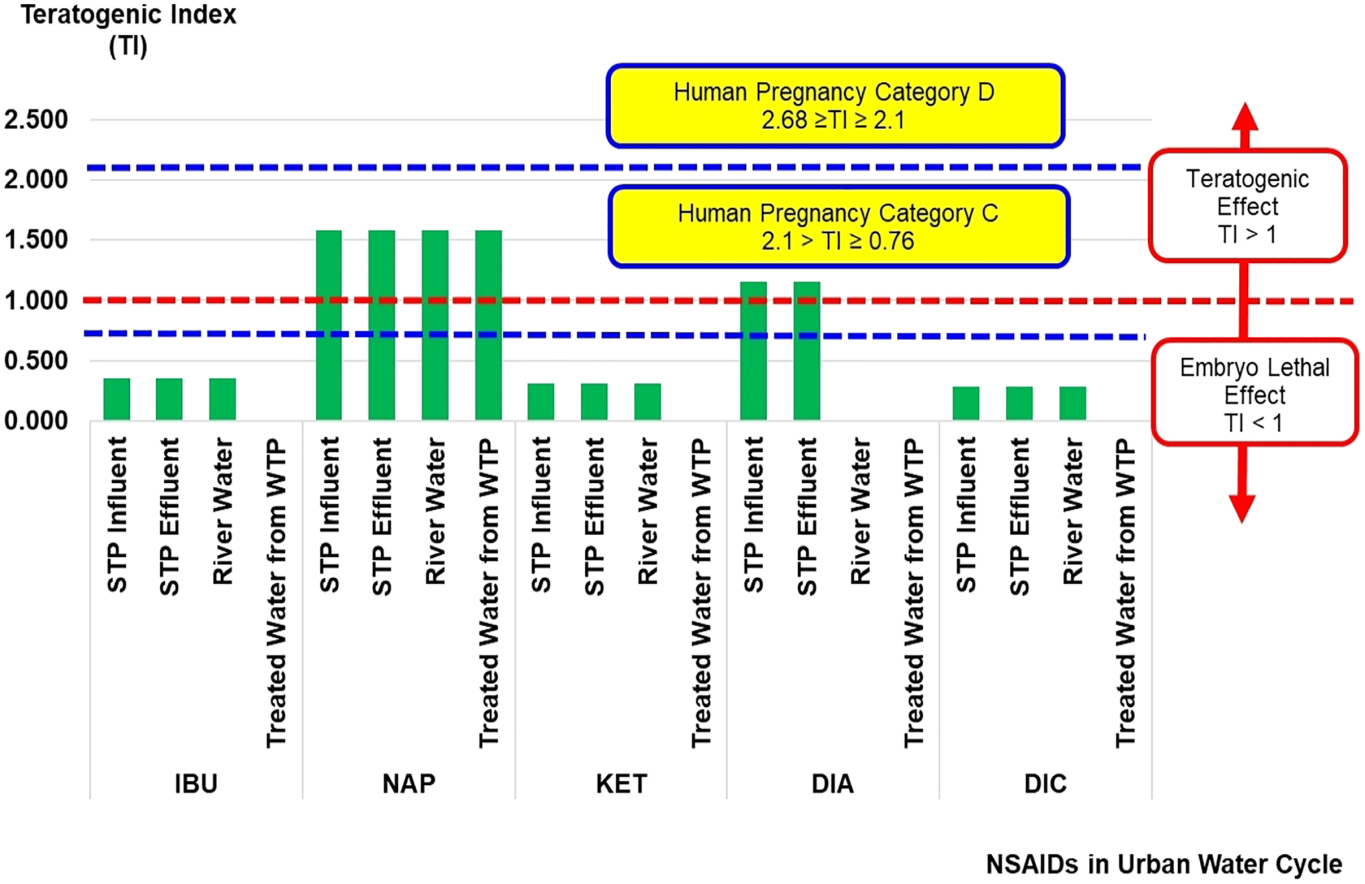
Teratogenic index (TI) of NSAIDs in the urban water cycle.

The teratogenic index (TI) of NSAIDs in the urban water cycle is shown in Fig. 3. A teratogen is any agent that can cause malformation or abnormality in foetal development during pregnancy. Teratogens are usually discovered after an increased prevalence of a particular congenital disability (District of Columbia Department of Health, 2010). The TI values can estimate the risk of teratogenic effects of contaminants on embryo development. The TI values presented in the current study were based on the risk quotient of LC_50_ over the risk quotient of EC_50_ (Eq. (3)) from aquatic organisms (fish, algae, crustaceans, and bacteria). The complete sets of risk quotient LC_50_ and EC_50_ achieved for NSAIDs (IBU, NAP, KET, DIA, and DIC) under study were from fish and crustaceans as summarised in Table 2.

In general, TI values of more than 1 indicate a teratogenic effect, while TI values of less than 1 indicate a lethal embryo effect. The TI values for NAP (influent STP: 1.58 × 10^0^, effluent STP: 1.58 × 10^0^, river water: 1.58 × 10^0^, and treated water: 1.58 × 10^0^), and DIA (influent STP: 1.16 × 10^0^, and effluent STP: 1.16 × 10^0^) in the urban water cycle were more than 1, indicating the possibilities of a teratogenic effect. The TI values for DIA in the surface and treated water samples were 0 × 10^0^ (not detected concentration of DIA). The other NSAIDs (IBU, KET, and DIC) had less than 1, indicating the possibilities of lethal embryo effects. The ranges of NAP and DIA concentrations recorded in the current study could induce an adverse effect on foetal (prenatal or embryonic period) development.

Lee et al. (2013) reported the TI values according to the pregnancy category by the United States Food and Drug Administration (US FDA) based on the effect of antileptic drugs in the *Danio rerio* embryo model (Lee et al., 2013). There are five US FDA pregnancy categories, namely categories A, B, C, D and X (Fisher et al., 1999). These categories are in ascending order based on the severity of toxicity effects of contaminants on embryo development during human pregnancy. Overall, the TI values of NAP and DIA can be classified as Human Pregnancy Category C (2.1 > TI ≥ 0.76).

The *Danio rerio* embryo development is close to the embryogenesis of higher vertebrates, including humans, making it ideal for studying the ecotoxicity of natural and synthetic contaminant(s) on embryonic development (Weigt et al., 2011). The types of lethal effects observed on the *Danio rerio* embryo model tested by Weigt et al. (2011) were coagulated egg (with no clear organ structures recognized) and no heartbeat. The types of teratogenic effects observed on the same model were malformation on the head, eyes, sacculi/otoliths (formation of no, one or more than two otoliths per sacculus as well as absence or abnormally shaped sacculi or vesicles), chords (entail malformation of the spinal cord), tail (malformation of the tail was assessed when the tail was bent), tail tip (it was assessed when the spike was bent or twisted), scoliosis, deformity of yolk, and growth retardation (it was assessed at three dpf (days post fertilisation) when the embryo had a body length below 2.8 mm) (Weigt et al., 2011).

**Figure 4:**
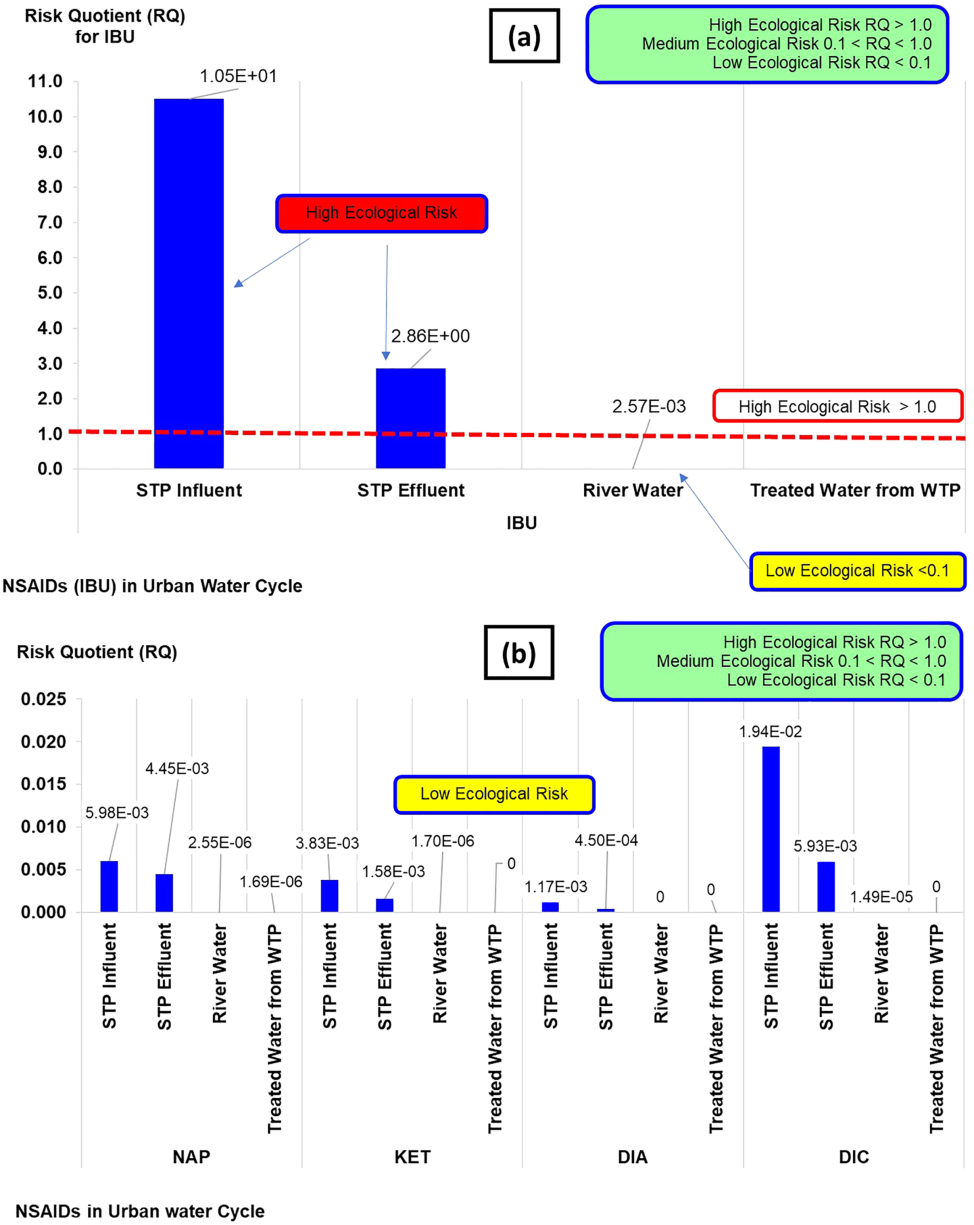
Risk quotient (RQ) of NSAIDs in the urban water cycle.

The risk quotient (RQ) of NSAIDs in the urban water cycle is shown in Fig. 4. The classifications of RQ are high (RQ > 1.0), medium (0.1 < RQ < 1.0), and low risk (RQ < 1). The RQ values obtained from the NSAIDs concentrations in the current study could be categorised as high and low risk. The high risk was from IBU concentration in influent STP (RQ: 1.05 × 10^1^) and the effluent STP (RQ: 2.86 × 10^0^), while the rest of the IBU (river water and treated water) and NAP, KET, DIA, and DIC could be categorised as low risk (RQ: ranging from 0 × 10^0^ to 1.90 × 10^−2^). Overall, the RQ indicated of a general ecological risk assessment (high and low risk) towards selected aquatic organisms. It does not give a detailed insight or explanation of the risk of contamination in aqueous environments when detailed as TI.

## Conclusions

The environmental fate of pharmaceutical residue in the urban water cycle was determined by the high concentration of NSAIDs in the influent STP ranging from 5.87 × 10^3^ to 7.18 × 10^4^ ng/L, showing that the primary source of contaminants comes from the sewage of urban areas. The percentage of NSAIDs in the urban water cycle gradually decreased, but river water and treated water was still contaminated with NSAIDs at the nanoscale ranging between 3.6 × 10^0^ and 3.06 × 10^1^ ng/L. The STPs could reduce the compounds from 25.6% to 92.3%. Positive linear correlations were observed in the influent *vs* effluent (0.994), effluent *vs* treated water (0.992), influent *vs* treated water (0.982), influent *vs* surface water (0.950), effluent *vs* surface water (0.921), and surface water *vs* treated water (0.888) models. Thus, good predictions on NSAIDs in the urban water cycle could be made on the basis of these models. This study illustrated the persistence of pharmaceutical products in the water processing cycle in reaching the community. Based on the degradation pathways of NSAIDs, there were possibilities of the metabolite presence in both surface and treated water. The actual effect on human health through foetal development and environmental risk could also pose a risk to human health, livestock production, and plantation. TI values for NAP and DIA were found to be higher than 1, giving the teratogenic effect, while RQ for IBU in the influent and the effluent of the STP was more than 1, classified as a high ecological risk.

The pathway of the pharmaceuticals from the STP to the treated water was significantly different, showing the ability of the treatment process in the STP to reduce certain pharmaceuticals from the domestic process through the current process up to a certain level and discharge them into the surface water. The dilution factor in the surface water and the river reclamation process reduced the concentration of pharmaceuticals in nature. However, but the TI and RQ assessments showed significant toxicity impacts based on NAP (STP influent and effluent, river water, and treated water) and DIA (STP influent and effluent) as TI > 1; IBU (STP influent and effluent) as RQ > 1 on the humans and environment. A prediction of the selected pharmaceuticals in the water cycle could be made on the basis of real-time quantification data for the occurrence of pharmaceutical residue in the water cycle. Therefore, it is essential to constantly monitor high-risk pharmaceuticals in the environment to protect human health, aquatic diversity, and the environment’s water health.

## Supplemental Information

10.7717/peerj.14719/supp-1 Supplemental Information 1 Raw data for Figures 3 and 4 and Table S-5.

10.7717/peerj.14719/supp-2 Supplemental Information 2 Supplemental Tables S-1 to S-3. Table S-1 Chemical structure and molecular formula of targeted compounds
Table S-2 Recovery and quality standard for targeted analyte
Table S-3. ANOVA analysis on NSAIDs in the urban water cycle

10.7717/peerj.14719/supp-3 Supplemental Information 3 Raw Data. Table R-1 Concentration and percentage differences of targeted compounds (Figure 3)
Table R-2 Raw data for Risk Quotient (RQ) (Figure 5) and Teratogenic Index (TI) (Figure 4)
Table R-3 Raw data for Pearson correlation (Table 4)

## Additional Information and Declarations

### Data Availability

The following information was supplied regarding data availability: Raw data is available in the Supplemental Files.
