# Supplementary material for: Determination and risk assessment of pharmaceutical residues in the urban water cycle in Selangor Darul Ehsan, Malaysia
Source: PeerJ. 2023 Feb 1;11:e14719. doi: 10.7717/peerj.14719 (PMC9899055; doi:10.7717/peerj.14719)
Supplement: Supplemental Information 2 — Table S-1 Chemical structure and molecular formula of targeted compounds Table S-2 Recovery and quality standard for targeted analyte Table S-3. ANOVA analysis on NSAIDs in the urban water cycle [file peerj-11-14719-s002.docx]

**Determination and Risk Assessment of Pharmaceutical Residues in the Urban Water Cycle**

Zarimah Mohd Hanafiah^1^, Wan Hanna Melini Wan Mohtar^1^, Teh Sabariah Abd Manan^2,3^, Nur Aina Bachi'^1^, Nurfaizah Abu Tahrim^4^, Haris Hafizal Abd Hamid^5^, Abdulnoor A. J. Ghanim^6^, Amirrudin Ahmad^2,7^, Nadiah Wan Rasdi^2,8^, Hamidi Abdul Aziz^3^

^1^Department of Civil Engineering, Faculty of Engineering and Built Environment, Universiti Kebangsaan Malaysia, 43600 UKM Bangi, Selangor Darul Ehsan, Malaysia.

^2^Institute of Tropical Biodiversity and Sustainable Development, Universiti Malaysia Terengganu, 21030 Kuala Nerus, Terengganu Darul Iman, Malaysia.

^3^School of Civil Engineering, Universiti Sains Malaysia, 14300 Nibong Tebal, Pulau Pinang, Malaysia.

^4^Department of Chemical Sciences, Faculty of Science and Technology, Universiti Kebangsaan, 43600 UKM Bangi, Selangor Darul Ehsan, Malaysia.

^5^Department of Earth Sciences and Environment, Faculty of Science and Technology, Universiti Kebangsaan Malaysia, 43600 UKM Bangi, Selangor Darul Ehsan, Malaysia.

^6^Department of Civil Engineering, College of Engineering, Najran University, Najran 61441, KSA.

^7^Faculty of Science and Marine Environment, Universiti Malaysia Terengganu, 21030 Kuala Nerus, Terengganu Darul Iman, Malaysia.

^8^Faculty of Fisheries and Food Science, Universiti Malaysia Terengganu, 21030 Kuala Nerus, Terengganu Darul Iman, Malaysia.

Corresponding Author:

Wan Hanna Melini Wan Mohtar^1^

Department of Civil Engineering, Faculty of Engineering and Built Environment, Universiti Kebangsaan Malaysia, 43600 UKM Bangi, Selangor Darul Ehsan, Malaysia.

Email address: hanna@ukm.edu.my

**Table S-1 Chemical structure and molecular formula of targeted compounds**

| Targeted compounds | Chemical Structure | Molecular formula | CAS number | Molecular Weight (g/mol) | LogK_ow_ |
| --- | --- | --- | --- | --- | --- |
| Ibuprofen (IBU) | 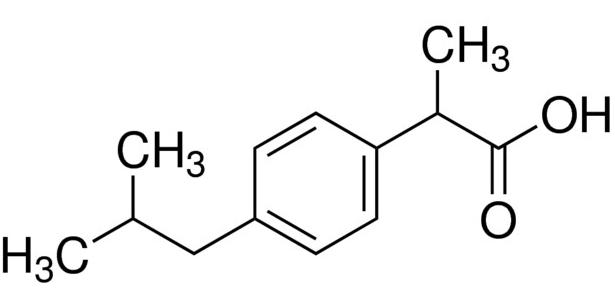 | C_13_H_18_O_2_ | 15687-27-1 | 206.28 | 3.97 |
| Naproxen (NAP) | 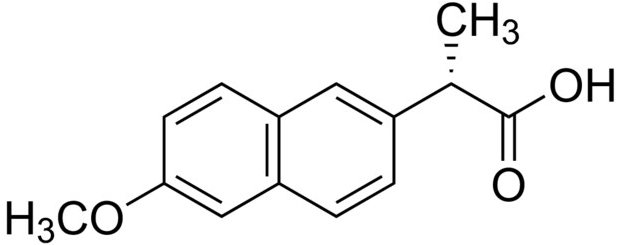 | C_14_H_14_O_3_ | 22204-53-1 | 230.26 | 3.18 |
| Ketoprofen (KET) | 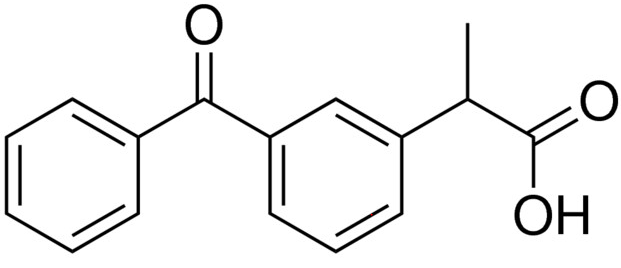 | C_16_H_14_O_3_ | 22071-15-4 | 254.28 | 2.66 |
| Diazepam (DIA) | 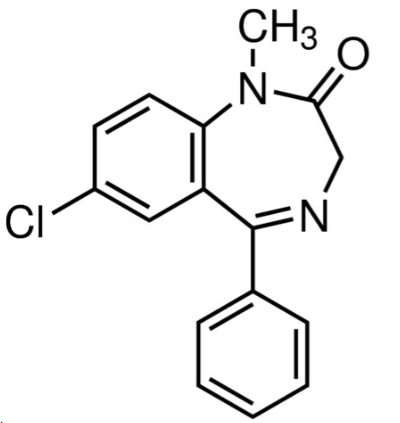 | C_16_H_13_ClN_2_O | 439-14-5 | 284.74 | 2.85 |
| Diclofenac (DIC) | 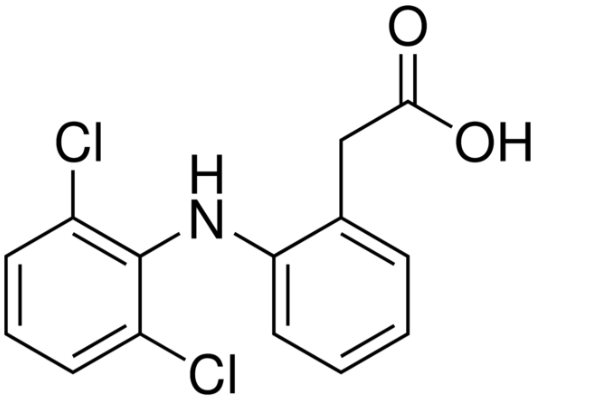 | C_14_H_11_Cl_2_NO_2_ | 15307-86-5 | 296.15 | 1.90 |

**Table S-2 Recovery and quality standard for targeted analyte**

| Analyte | Retention  time (min) | Recovery (%) | Linearity from response | Coefficient of determination (*R^2^*) from a response | Coefficient of variation (σ/mean)  (%) | Standard deviation of response (σ) | Slope of the calibration curve  (s) | LOD  (x10^5^ ng/L)  3.3*(σ/s) | LOQ  (x10^6^ ng/L)  10*(σ/s) |
| --- | --- | --- | --- | --- | --- | --- | --- | --- | --- |
| IBU | 9.05 | 99.1 | y=53977x-21811 | 0.9844 | 15.14 | 7093.08 | 53977 | 4.337 | 1.314 |
| NAP | 12.0 | 97.7 | y=4331.4x+6382.8 | 0.9607 | 5.32 | 505.919 | 4331.4 | 3.854 | 1.168 |
| KET | 12.8 | 111.7 | y=106608x+65246 | 0.9889 | 16.30 | 21820.06 | 106608 | 6.754 | 2.046 |
| DIA | 14.9 | 74.86 | y=144164x+162864 | 0.9755 | 17.59 | 29990.55 | 144164 | 6.865 | 2.080 |
| DIC | 13.0 | 69.1 | y=48855x+6961.8 | 0.9992 | 9.36 | 5241.51 | 48855 | 3.540 | 1.072 |

**Table S-3 The pharmaceutical residue (IBU, NAP, KET, DIA, DIC) in the urban water**

| **Country** | **Water cycle** | **Pharmaceuticals** | **Ref.** |
| --- | --- | --- | --- |
| Sweden | STP | IBU, NAP, KET, DIC | (Larsson et al., 2014) |
| Canada | STP | IBU, NAP | (Guerra et al., 2014) |
| South Africa | STP | IBU, NAP, DIC | (Madikizela and Chimuka, 2017) |
| Spain | STP | IBU, NAP, KET, DIC | (Gracia-Lor et al., 2017) |
| Spain | Surface water | IBU, KET, DIC | (Jurado et al., 2021) *Groundwater |
| Spain | Treated water | IBU, NAP, DIC | (Carmona et al., 2014) |
| India | STP | DIA | (Subedi et al., 2017) |
| Beijing, China | STP | DIA | (Wang et al., 2017) |
| United Kingdom | STP | DIA | (Baker and Kasprzyk-Hordern, 2013) |
| Thailand | Surface water | IBU, NAP, DIC | (Tewari et al., 2013) |
| Italy | Surface water | IBU, NAP, KET, DIC | (Marchese et al., 2003) |
| Italy | Surface water | DIA | (Zuccato et al., 2000) |
| Serbia | Treated water | KET | (Petrović et al., 2014) |
| France | Treated water | IBU, NAP, DIC | (Togola and Budzinski, 2008) |
| Algeria | Treated water | KET | (Kermia et al., 2016) |
| Johor, Malaysia | STP | IBU, NAP | (Yacob et al., 2017) |
| Selangor, Malaysia | Surface water | KET | (Abu Tahrim et al., 2018) |
| Putrajaya, Malaysia | Treated water | DIC | (Wee et al., 2020) |
| Selangor, Malaysia | STP, Surface water, Treated water | IBU, NAP, KET, DIA, DIC | Current Study |

**cycle (STP, surface water, and treated water).**

| **Model No.** | **ANOVA Model** | | **Sum of Squares** | **df** | **Mean Square** | **F** | **Sig. F Change** |
| --- | --- | --- | --- | --- | --- | --- | --- |
| 1. | Influent Vs. Effluent | SSW | 1888825833 | 1 | 1888825833 | 235.671 | 0.001 |
|  |  | SSB | 24043967.36 | 3 | 8014655.785 |  |  |
|  |  | SST | 1912869800 | 4 |  |  |  |
| a Dependent Variable: Effluent | | | |  |  |  |  |
| b Predictors: (Constant), Influent | | | |  |  |  |  |
| 2. | Effluent Vs. Surface Water | SSW | 484.843 | 1 | 484.843 | 16.746 | 0.026 |
|  |  | SSB | 86.856 | 3 | 28.952 |  |  |
|  |  | SST | 571.699 | 4 |  |  |  |
| a Dependent Variable: Water | | | |  |  |  |  |
| b Predictors: (Constant), Effluent | | | |  |  |  |  |
| 3. | Surface Water Vs. Treated Water | SSW | 244.891 | 1 | 244.891 | 11.223 | 0.044 |
|  |  | SSB | 65.464 | 3 | 21.821 |  |  |
|  |  | SST | 310.354 | 4 |  |  |  |
| a Dependent Variable: Treated water | | | |  |  |  |  |
| b Predictors: (Constant), River water | | | |  |  |  |  |

**Table S-4 ANOVA analysis on NSAIDs in the urban water cycle.**

**Table S-5 The Pearson correlation on NSAIDs in the urban water cycle**

| **Pearson Correlation** | **Influent** | **Effluent** | **Surface Water** | **Treated Water** |
| --- | --- | --- | --- | --- |
| Influent | 1.000 | - | - | - |
| Effluent | 0.994** | 1.000 | - | - |
| Surface Water | 0.950* | 0.921 | 1.000 | - |
| Treated Water | 0.982** | 0.992** | 0.888* | 1.000 |
| ** Correlation is significant at the 0.01 level (2-tailed).  * Correlation is significant at the 0.05 level (2-tailed). | | | | |
