# Supplementary material for: Determination and risk assessment of pharmaceutical residues in the urban water cycle in Selangor Darul Ehsan, Malaysia
Source: PeerJ. 2023 Feb 1;11:e14719. doi: 10.7717/peerj.14719 (PMC9899055; doi:10.7717/peerj.14719)
Supplement: Supplemental Information 3 — Table R-1 Concentration and percentage differences of targeted compounds (Figure 3) Table R-2 Raw data for Risk Quotient (RQ) (Figure 5) and Teratogenic Index (TI) (Figure 4) Table R-3 Raw data for Pearson correlation (Table 4) [file peerj-11-14719-s003.docx]

**Determination and Risk Assessment of Pharmaceutical Residues in the Urban Water Cycle**

Zarimah Mohd Hanafiah^1^, Wan Hanna Melini Wan Mohtar^1^, Teh Sabariah Abd Manan^2,3^, Nur Aina Bachi'^1^, Nurfaizah Abu Tahrim^4^, Haris Hafizal Abd Hamid^5^, Abdulnoor A. J. Ghanim^6^, Amirrudin Ahmad^2,7^, Nadiah Wan Rasdi^2,8^, Hamidi Abdul Aziz^3^

^1^Department of Civil Engineering, Faculty of Engineering and Built Environment, Universiti Kebangsaan Malaysia, 43600 UKM Bangi, Selangor Darul Ehsan, Malaysia.

^2^Institute of Tropical Biodiversity and Sustainable Development, Universiti Malaysia Terengganu, 21030 Kuala Nerus, Terengganu Darul Iman, Malaysia.

^3^School of Civil Engineering, Universiti Sains Malaysia, 14300 Nibong Tebal, Pulau Pinang, Malaysia.

^4^Department of Chemical Sciences, Faculty of Science and Technology, Universiti Kebangsaan, 43600 UKM Bangi, Selangor Darul Ehsan, Malaysia.

^5^Department of Earth Sciences and Environment, Faculty of Science and Technology, Universiti Kebangsaan Malaysia, 43600 UKM Bangi, Selangor Darul Ehsan, Malaysia.

^6^Department of Civil Engineering, College of Engineering, Najran University, Najran 61441, KSA.

^7^Faculty of Science and Marine Environment, Universiti Malaysia Terengganu, 21030 Kuala Nerus, Terengganu Darul Iman, Malaysia.

^8^Faculty of Fisheries and Food Science, Universiti Malaysia Terengganu, 21030 Kuala Nerus, Terengganu Darul Iman, Malaysia.

Corresponding Author:

Wan Hanna Melini Wan Mohtar^1^

Civil Engineering, Faculty of Engineering and Built Environment, Universiti Kebangsaan Malaysia, 43600 UKM Bangi, Selangor Darul Ehsan, Malaysia.

Email address: hanna@ukm.edu.my

**Table R-1 Raw data for Risk Quotient (RQ) (Figure 4) and Teratogenic Index (TI) (Figure 3)**

| Targeted compounds | Urban Water Cycle | Concentration (ng/L) | Concentration (mg/L) | RQ LC50 | RQEC50 | TI | RQ |
| --- | --- | --- | --- | --- | --- | --- | --- |
| IBU | STP Influent | 14700 | 0.0147 | 1.824 | 5.158 | 0.354 | 10.5 |
|  | STP Effluent | 4000 | 0.004 | 0.496 | 1.404 | 0.354 | 2.86 |
|  | Surface Water | 3.6 | 0.0000036 | 0.000 | 0.00126 | 0.354 | 0.0026 |
|  | Treated Water from WTP | 0 | 0 | 0.000 | 0.000 | 0.000 | 0.000 |
| NAP | STP Influent | 71800 | 0.0718 | 0.000653 | 0.000413 | 1.58 | 0.00598 |
|  | STP Effluent | 53370 | 0.05337 | 0.000485 | 0.000307 | 1.58 | 0.00445 |
|  | Surface Water | 30.63 | 0.00003063 | 0.000000278 | 0.000000176 | 1.58 | 0.00000255 |
|  | Treated Water from WTP | 20.33 | 0.00002033 | 0.000000185 | 0.000000117 | 1.58 | 0.00000169 |
| KET | STP Influent | 23930 | 0.02393 | 0.00392 | 0.0125 | 0.313 | 0.00383 |
|  | STP Effluent | 9900 | 0.0099 | 0.00162 | 0.00518 | 0.313 | 0.00158 |
|  | Surface Water | 10.6 | 0.0000106 | 0.00000173 | 0.00000555 | 0.313 | 0.00000170 |
|  | Treated Water from WTP | 4.4 | 0.0000044 | 0.000 | 0.000 | 0.000 | 0.000 |
| DIA | STP Influent | 5870 | 0.00587 | 0.000481 | 0.000416 | 1.16 | 0.00117 |
|  | STP Effluent | 450 | 0.00045 | 0.0000369 | 0.0000319 | 1.16 | 0.000450 |
|  | Surface Water | 0 | 0 | 0.000 | 0.000 | 0.00 | 0.000 |
|  | Treated Water from WTP | 0 | 0 | 0.000 | 0.000 | 0.00 | 0.000 |
| DIC | STP Influent | 19400 | 0.0194 | 0.000242 | 0.000865 | 0.280 | 0.0194 |
|  | STP Effluent | 5930 | 0.00593 | 0.0000740 | 0.000264 | 0.280 | 0.00593 |
|  | Surface Water | 14.85 | 0.00001485 | 0.000000185 | 0.000000662 | 0.280 | 0.0000149 |
|  | Treated Water from WTP | 0 | 0 | 0.000 | 0.000 | 0.000 | 0.000 |

**Table R-2 Raw data for Pearson correlation (Table S-5)**

|  |  | Influent | Effluent | Surface_water | Treated_water |
| --- | --- | --- | --- | --- | --- |
| Influent | Pearson Correlation | 1 | .994** | .950* | .982** |
|  | Sig. (2-tailed) |  | 0.001 | 0.013 | 0.003 |
|  | N | 5 | 5 | 5 | 5 |
| Effluent | Pearson Correlation | .994** | 1 | .921* | .992** |
|  | Sig. (2-tailed) | 0.001 |  | 0.026 | 0.001 |
|  | N | 5 | 5 | 5 | 5 |
| Surface_water | Pearson Correlation | .950* | .921* | 1 | .888* |
|  | Sig. (2-tailed) | 0.013 | 0.026 |  | 0.044 |
|  | N | 5 | 5 | 5 | 5 |
| Treated_water | Pearson Correlation | .982** | .992** | .888* | 1 |
|  | Sig. (2-tailed) | 0.003 | 0.001 | 0.044 |  |
|  | N | 5 | 5 | 5 | 5 |
| ** Correlation is significant at the 0.01 level (2-tailed). | | | | | |
| * Correlation is significant at the 0.05 level (2-tailed). | | | | | |
